# Validation of Somno-Art Software, a novel approach of sleep staging, compared with polysomnography in disturbed sleep profiles

**DOI:** 10.1093/sleepadvances/zpab019

**Published:** 2021-12-09

**Authors:** Laurie Thiesse, Luc Staner, Patrice Bourgin, Thomas Roth, Gil Fuchs, Debora Kirscher, Jean-Yves Schaffhauser, Jay B Saoud, Antoine U Viola

**Affiliations:** 1 PPRS, Colmar, France; 2 Unité d’Exploration des Rythmes Veille-Sommeil, Centre Hospitalier de Rouffach, Rouffach, France; 3 Sleep Disorders Center and CIRCSom (International Research Center for ChronoSomnology), Strasbourg University Hospital, Strasbourg, France; 4 CNRS UPR 3212, Institute for Cellular and Integrative Neurosciences, Strasbourg, France; 5 Sleep Disorders Center, Henry Ford Hospital, Detroit, MI, USA; 6 PPRS Research Inc., Groton, MA, USA; 7 PPDA, LLC, Groton, MA, USA

**Keywords:** automatic sleep analysis, heart rate, actimetry, obstructive sleep apnea, insomnia, major depressive disorder

## Abstract

**Study Objectives:**

Integrated analysis of heart rate (electrocardiogram [ECG]) and body movements (actimetry) during sleep in healthy subjects have previously been shown to generate similar evaluation of sleep architecture and continuity with Somno-Art Software compared to polysomnography (PSG), the gold standard. However, the performance of this new approach of sleep staging has not yet been evaluated on patients with disturbed sleep.

**Methods:**

Sleep staging from 458 sleep recordings from multiple studies comprising healthy and patient population (obstructive sleep apnea [OSA], insomnia, major depressive disorder [MDD]) was obtained from PSG visual scoring using the American Academy of Sleep Medicine rules and from Somno-Art Software analysis on synchronized ECG and actimetry.

**Results:**

Inter-rater reliability (IRR), evaluated with 95% absolute agreement intra-class correlation coefficient, was rated as “excellent” (ICC_AAAvg95%_ ≥ 0.75) or “good” (ICC_AAAvg95%_ ≥ 0.60) for all sleep parameters assessed, except non-REM (NREM) and N3 sleep in healthy participants (ICC_AAAvg95%_ = 0.43, ICC_AAAvg95%_ = 0.56) and N3 sleep in OSA patients (ICC_AAAvg95%_ = 0.59) rated as “fair” IRR. Overall sensitivity, specificity, accuracy, and Cohen’s kappa coefficient of agreement (κ) on the entire sample were respectively of 93.3%, 69.5%, 87.8%, and 0.65 for wake/sleep classification and accuracy and κ were of 68.5% and 0.55 for W/N1+N2/N3/rapid eye movement (REM) classification. These performances were similar in healthy and patient population.

**Conclusions:**

The present results suggest that Somno-Art can be a valid sleep-staging tool in both healthy subjects and patients with OSA, insomnia, or MDD. It could complement existing non-attended techniques measuring sleep-related breathing patterns or be a useful alternative to laboratory-based PSG when this latter is not available.

**Statement of Significance**

The development of wearable devices and algorithms to monitor and stage sleep in long-term or ambulatory settings is rising. However, most of the devices on the market today lack robust validation studies, especially in patient populations, and thus cannot be considered as good and reliable alternative to the gold standard, polysomnography. This validation study of an automatic cardiac and movement-based sleep scoring algorithm shows promise as a valuable aid for diagnosis and treatment-follow-up of sleep disorders and disturbances in the patients’ living environment.

## Introduction

Polysomnography (PSG) is the gold standard for objective sleep monitoring and the diagnosis of many sleep disorders. PSG, composed mainly of an electroencephalogram (EEG), an electro-oculogram (EOG), and an electro-myogram (EMG), is cumbersome and time-consuming to set up and is therefore costly and with limited access (long waiting lists and some large geographical areas are poorly equipped). For these reasons, PSG is generally limited to a maximum of one or two recording nights in the sleep laboratory. In parallel, the evaluation of sleep architecture and continuity consists in the visual scoring of 30-sec epochs PSG recordings based on the standard adopted by the health care institutions, the American Academy of Sleep Medicine (AASM) manual [1]. Visual scoring is a tedious task and several studies reported an inter-rater reliability (IRR) under 85% [2–5].

Therefore, the development of new technologies to respond to these limitations of PSG could facilitate and improve clinical evaluation of sleep disturbances. Indeed, insomnia is often diagnosed based only on nonobjective tools such as the clinical interview, questionnaires, or sleep diary which are much easier to obtain than PSG. Even if these nonobjective tools are useful and necessary to guide the diagnosis, objective sleep monitoring is mandatory to detect potential associated sleep disorders and may deliver information not inherent in the subjective patient report such as detecting sleep state misperception. Furthermore, nonobjective tools often overestimate the symptoms compared to objective measures [6]. The diagnosis of insomnia, therefore, would benefit from several successive recording nights to be reliably evaluated. In addition, at-home sleep recording would avoid confounding factors specific to the sleep laboratory settings such as the first night effect, and reflect more accurately the normal environment in which the patient is living [7–9]. Diagnosis of sleep apnea syndrome would also benefit from an ambulatory sleep staging system to supplement ambulatory respiratory polygraphy that does not discriminate between wake and sleep states, leading to misestimation of total sleep time (TST) and therefore of the apnea-hypopnea index (AHI). A new wave of research focuses on the detection of sleep independently of brain electrical activity (EEG), in adopting a multisensory approach based on the knowledge that autonomic variables such as heart rate and its variability are sleep stage-dependent [10–12]. However, most of the wearable devices on the market today lack of robust validation studies and cannot be considered as good and reliable alternatives to PSG [13]. In 2016, Muzet et al. validated in healthy volunteers the Somno-Art Software against PSG [14]. Somno-Art Software evaluates sleep architecture and continuity from an integrated analysis of heart rate and body movements. This study found an excellent intra-class correlation (according to Cicchetti [15] cutoffs) between Somno-Art Software and PSG for the combination of 12 sleep architecture and continuity descriptors (i.e. sleep efficiency [SE], sleep latency [SL], REM sleep) useful for the clinician in the diagnosis and quantification of treatments (both pharmacological and interventional).

Sleep disorders such as insomnia or obstructive sleep apnea (OSA) affect sleep architecture and continuity, complicating the visual scoring [2, 3, 16] and leading to lower IRR compared to healthy adults [2, 3, 17]. Therefore, most of the wearable devices based on cardiac and body movement or EEG signals are so far exclusively or mostly validated in healthy populations [18–20].

The aim of the data presented here is to evaluate the performance of the new approach of sleep staging of Somno-Art Software based on heart rate and body movement, on disturbed sleep architecture and continuity. To do so, sleep recordings coming from healthy subjects and patients suffering from OSA, insomnia, or major depressive disorder (MDD) were analyzed. It is hypothesized that Somno-Art Software performances on healthy and pathological populations will be similar.

## Methods

### Dataset

#### Source studies.

The dataset used for this research is based on data collected from six studies.

Recording nights from healthy subjects were acquired from two studies. Study 1 primary objective was to investigate relationship between daytime activity and night sleep structure and the impact of noise on sleep patterns. Study 2 primary objective was to investigate the effect of light on sleep, wake, EEG, and cognitive performances as a function of homeostatic sleep drive. All recorded nights from these two studies were included in the dataset.

Recording nights from patients were acquired from four studies.—The OSA study included patients diagnosed with OSA syndrome—The insomniac study and the two depression studies’ primary objectives were to evaluate the efficacy, safety, and tolerability of investigational drugs. Only pretreatment nights were included in the dataset. For all studies included in the present analysis and before undergoing sleep recordings, a standard screening of patients and healthy subjects’ health status was done. More information on the protocol descriptions are detailed in Supplementary 1.

All study protocols were approved by institutional review boards in accordance with the Declaration of Helsinki and the guidelines on Good Clinical Practice. Written consent was obtained from all participants according to local requirements.

#### Participants.

All subjects were free of any drug or medication that could affect sleep. Patients were diagnosed with OSA based on the AHI (≥5 [21]). Insomnia was diagnosed with the Insomnia Severity Index (> 15). MDD patients fulfilled diagnostic and statistical manual-4 or -5 criteria (using MINI 6.0 or 7.0) and had a score ≥ 30 on the Inventory of Depressive Symptomatology (IDS-C30) or on the Montgomery-Åsberg Depression Rating Scale (MADRS) and a score ≥ 4 (markedly ill or worse) on the Clinical Global Impressions Severity Scale (CGI-S).

From a pool of 509 nights from 267 subjects, 458 recording nights from 246 subjects were included in the dataset after removing recordings that could not be analyzed due to Somno-Art Software limitations: recording nights with a time in bed under 5 h, recording nights with periodic movements, or recording nights with long R–R signal loss. In total 79 nights from 26 healthy participants (up to five nights/subject), 33 nights from 30 patients with OSA (up to two nights/subject), 135 nights from 66 patients with insomnia (up to three nights/subject), and 211 nights from 124 patients with MDD (up to two nights/subject) were included in the analysis. Other demographic and baseline information of each study group are presented in Table 1.

**Table 1.** Demographic data per study group

	Raw dataset		Final dataset					
Study group	Subjects *N*	Nights *N*	Subjects *N*	Nights *N*	Data loss* %	Age Mean ± *SD*	F/M ratio	AHI Mean ± *SD*
Healthy	26	83	26	79	4.82	25 ± 5.8	13/13	NA
OSA patient	36	39	30	33	15.38	54 ± 14	12/18	23 ± 18
Insomniac	68	150	66	135	10.00	44 ± 14	44/22	NA
Depressed	137	237	124	211	10.97	46 ± 13	83/41	NA
All subjects	267	509	246	458	10.02	44 ± 15	152/94	NA

Number of subjects and nights for the raw and the final dataset, mean (±*SD*) age, and female/male (F/M) ratio of subjects by study, mean (±*SD*) apnea-hypopnea index (AHI) of the OSA group.

NA, not available.

*Recording nights not analyzable by Somno-Art Software.

### Study design

All the recordings combined standard PSG with ECG and actimetry recordings.

#### PSG.

Multiple PSG recording systems were used in the various studies (Compumedics ProFusion PSG 3; Compumedics Siesta 802a [Compumedics, Abbotsford, Australia]) but all had at least six EEG derivations (C3-A2, C4-A1, F3-A2, F4-A1, O1-A2, O2-A1), two EOG electrodes, two chin EMG, and two ECG electrodes. All PSG recorded data were converted into European Data Format to be processed on a computer screen for visual analysis and scoring [22].

Sleep staging was performed according to the AASM rules and the resulting reference classes were obtained by combining N1 and N2 into a single “N1 + N2” class while the remaining classes (wake, N3, and REM) were unchanged. The nights from the healthy and the OSA subjects were scored by experienced scorers, 1 per study. The insomnia and the depression studies were scored by an independent expert scorer of the Siesta Group (Vienna, Austria) using the computer-assisted Somnolyzer software [23].

#### Cardiac activity from ECG.

Cardiac beats position was extracted from the PSG ECG lead with Medilog Darwin v2.8. To avoid misdetection, periods without signal were excluded, no other beat correction were applied (artifacts and ectopic beats were left as is). Successive inter-beats intervals (R–R intervals) were then computed from this continuous series of beats. Heart rate data were calculated from R–R intervals as HR = 60/RR (in seconds) and then interpolated at 1 Hz.

#### Wrist movement from actimetry.

Nondominant wrist movement activity was recorded using ActiGraph (Actigraph LLC, Pensacola, FL) activity monitor. Raw data were filtered and accumulated every second. The wrist actimetry was measured through the vector magnitude of accelerations obtained every second in the three dimensions of the space and its value is given in counts per second.

#### Somno-Art Software.

To perform Somno-Art Software 2.6.0 [3.1.0] analysis, a precise synchronization of the actimetry and the PSG ECG signal was achieved. A visual inspection to confirm that some occurring events such as cardiac arousals (sudden increase in heart rate followed by a return to initial values) were associated with wrist movements was performed.

Using heart rate at a beat-to-beat resolution and actimetry data at a 1 Hz resolution, sleep stage classification (wake, N1+N2, N3, REM) was performed at a 1-s epoch resolution. The latter 1-s epoch classification was merged into 30-s epochs to be compared to visual scoring. To do so, the more prevalent stage, or the first occurring stage when equally represented, was selected.

The sleep classification algorithm is based on the detection and quantification of physiological events such as movements or cardiac arousals in association with Support Vector Machine (SVM) detectors. SVM detectors were trained on a pool of recording nights (3 to 5 recordings, depending on the detector), optimized on 123 recording nights, and tested on a pool of 118 recordings. In a final step, the sleep stage classification is fine-tuned by more than 40 expert rules to better discriminate transition phases. More information on the data processing methodology is described in Muzet et al. [14].

### Statistical analysis

Based on the guidelines edited in *SLEEP* after the 2018 international biomarkers workshop on wearables in sleep and circadian science, recommended statistical tools described in Table 3*: Guidelines for performing and interpreting results from device validation of sleep and circadian metrics* (descriptive statistics, Bland-Altman plot, epoch-by-epoch (EBE) analysis [sensitivity, specificity, confusion matrix]), were used to evaluate the agreement of the Somno-Art Software to PSG [13].

#### Sleep parameter analysis.

Derived from the sleep stage classification, the following AASM sleep-wake statistics were computed: TST, SE, wake after sleep onset (WASO), and SL. In addition, latency to persistent sleep (LPS), defined as the elapsed time between lights-off and the first occurrence of continuous 10 min in any sleep stage, and REM sleep latency (REML), defined as the elapsed time between sleep onset and the first occurrence of REM sleep were computed.

To take into consideration the multiple nights from the same subject, the mean sleep parameters of each subject were calculated and only one data point per subject was used for the analysis.

The IRR between Somno-Art Software and the visual scorer was assessed for all sleep parameters (TST, SE, WASO, SL, LPS, REML, wake, N1 + N2, N3, NREM, and REM sleep) in calculating absolute agreement intra-class correlation coefficient (ICC_AAAvg_: the degree of absolute agreement for measurements) using two-way mixed model with “subject” as a random effect and “rater” as a fixed effect [24]. 95% ICC_AAAvg_ were estimated after 5% outlier data trimming (based on PSG visual scorer versus Somno-Art Software differences) procedure using 2.5% two-sided approach. An ICC estimate of 1 indicates perfect agreement and 0 indicates only random agreement (values increase by one method and decrease by another method, nondirectional). Cicchetti [15], provides commonly cited cutoffs for qualitative ratings of agreement based on ICC values 0–0.39: “poor” agreement; 0.40–0.59: “fair” agreement; 0.60–0.74: “good” agreement; 0.75–1: “excellent” agreement.

Bland-Altman plots were constructed to qualitatively assess the concordance between Somno-Art Software and the visual scorer and evaluate overall device performance. To quantify the bias, ±95% CI and the lower and upper agreement limits of the Bland-Altman, Design 3 of the NCSS software, which addresses multiple variables within-subject assessments, was used (https://www.ncss.com/wp-content/themes/ncss/pdf/Procedures/NCSS/Bland-Altman_Plot_and_Analysis.pdf). In short, the mean difference corresponds to the mean of the means and limits of agreement (LoA) calculation to the *SD* of a difference that considers pooled estimates of the within-subject and between-subject random errors, and the harmonic mean of the replicate counts. Finally, confidence interval estimation for LoA is based on the MOVER method, which provides adjusted confidence intervals and is accurate for small to moderate sample sizes. The Bland-Altman plots allow the visualization of discrepancies and the interpretation of biases: a positive bias indicates that the Somno-Art Software underestimated the observed outcome while a negative bias indicates that the Somno-Art Software overestimated the observed outcome.

#### EBE analysis.

Sensitivity, specificity, accuracy, and Cohen’s kappa coefficient of agreement κ [25] were used to evaluate EBE agreement. Sensitivity is defined as the ability to correctly classify PSG sleep epochs, while specificity is defined as the ability to correctly classify wake epochs. Accuracy indicates the percentage of epochs correctly labeled relative to PSG. κ indicates the agreement between the two hypnograms corrected for agreement due to chance. These metrics were computed on each night before evaluating the distribution on the whole dataset. The κ score scale was applied for evaluating agreement between recorders: <0: poor; 0–0.20: slight; 0.21–0.40: fair; 0.41–0.60: moderate; 0.61–0.80: substantial; 0.81–1: almost perfect agreement [25].

Confusion matrices represent EBE analysis by cross-tabulating the agreement and disagreement between Somno-Art Software and PSG visual scoring.

## Results

Table 2 presents the mean ± *SD* of each sleep architecture and continuity descriptors obtained with Somno-Art Software and visual scoring of PSG on the mean value of each subject (*n* = 246).

**Table 2.** Intra-class correlation coefficient between Somno-Art Software and PSG visual scoring

Subdivided in healthy and pathologies												
	All (*n* = 246)				Healthy (*n* = 26)				Pathologies (*n* = 220)			
	PSG (mean ± SD)	Somno-Art (mean ± SD)	ICC_95%AAAvg_ (LowB)		PSG (mean ± SD)	Somno-Art (mean ± SD)	ICC_95%AAAvg_ (LowB)		PSG (mean ± SD)	Somno-Art (mean ± SD)	ICC_95%AAAvg_ (LowB)	
TST (min)	368.5 ± 72.8	378.6 ± 69	0.91	(0.88)	434.9 ± 26.2	426.9 ± 25.6	0.88	(0.59)	360.6 ± 72.6	372.9 ± 70.3	0.91	(0.87)
SE (%)	76.6 ± 14.5	78.7 ± 13.3	0.90	(0.87)	90.7 ± 5.5	89 ± 5.4	0.88	(0.60)	75 ± 14.4	77.5 ± 13.5	0.89	(0.85)
WASO (min)	73.6 ± 49.8	65.9 ± 42	0.84	(0.79)	33.3 ± 20.9	41.7 ± 23.8	0.87	(0.58)	78.4 ± 50	68.8 ± 42.8	0.82	(0.76)
SL (min)	37.5 ± 37.4	35 ± 39.2	0.92	(0.89)	11.4 ± 10.4	11.1 ± 7.6	0.94	(0.86)	40.6 ± 38.2	37.8 ± 40.5	0.90	(0.87)
LPS (min)	48.1 ± 41.4	35.8 ± 39.4	0.88	(0.76)	15.7 ± 13.5	11.3 ± 7.7	0.81	(0.55)	51.9 ± 41.9	38.7 ± 40.7	0.86	(0.73)
REM L (min)	105.4 ± 55.5	98.9 ± 47.3	0.76	(0.69)	80.7 ± 40	80.9 ± 29.6	0.9	(0.85)	108.4 ± 56.4	101.1 ± 48.7	0.75	(0.66)
Wake (min)	112.4 ± 69.3	102.2 ± 63.1	0.90	(0.86)	44.7 ± 26.3	52.8 ± 25.8	0.88	(0.60)	120.3 ± 68.5	108 ± 63.7	0.89	(0.84)
N1 + N2 (min)	217.7 ± 58.2	222.9 ± 51.1	0.82	(0.77)	235.2 ± 43.9	225 ± 28.8	0.71	(0.36)	215.6 ± 59.5	222.7 ± 53.2	0.84	(0.78)
N3 (min)	74.9 ± 46.2	77 ± 29.3	0.63	(0.53)	106.8 ± 61.2	98.3 ± 19.8	0.56	(−0.03)	71.1 ± 42.7	74.5 ± 29.3	0.64	(0.53)
NREM (min)	292.6 ± 59.4	300 ± 55.1	0.87	(0.83)	342 ± 32.5	323.3 ± 20.7	0.43	(−0.16)	286.8 ± 59.1	297.2 ± 57.3	0.87	(0.82)
REM (min)	75.9 ± 28.1	78.6 ± 24.8	0.82	(0.77)	92.9 ± 22.6	103.6 ± 17.1	0.62	(0.14)	73.9 ± 28	75.7 ± 23.9	0.82	(0.76)
Pathologies subdivided in OSA, insomniac, and MDD patients												
	OSA (*n* = 30)				Insomniac (*n* = 66)				MDD (*n* = 124)			
	PSG (mean ± SD)	Somno-Art (mean ± SD)	ICC_95%AAAvg_ (LowB)		PSG (mean ± SD)	Somno-Art (mean ± SD)	ICC_95%AAAvg_ (LowB)		PSG (mean ± SD)	Somno-Art (mean ± SD)	ICC_95%AAAvg_ (LowB)	
TST (min)	377.1 ± 83.1	383.8 ± 105.1	0.90	(0.79)	348.4 ± 62.9	358.1 ± 54.9	0.90	(0.83)	363.1 ± 74.2	378.2 ± 66.5	0.90	(0.84)
SE (%)	75.3 ± 11.4	76.2 ± 14.7	0.74	(0.45)	72.8 ± 13	74.9 ± 11.4	0.90	(0.83)	76 ± 15.6	79.2 ± 14	0.90	(0.84)
WASO (min)	91.5 ± 59.6	84.6 ± 61.4	0.82	(0.60)	83.2 ± 45.8	78.3 ± 37.3	0.84	(0.73)	72.6 ± 49.2	59.7 ± 37.9	0.83	(0.73)
SL (min)	32 ± 34.6	32.2 ± 36.1	0.64	(0.22)	46.8 ± 37.4	41.9 ± 31.4	0.95	(0.92)	39.3 ± 39.2	37 ± 45.5	0.90	(0.86)
LPS (min)	48.4 ± 35.6	34.1 ± 36.8	0.66	(0.26)	58.1 ± 39	42.6 ± 32.2	0.90	(0.65)	49.4 ± 44.7	37.7 ± 45.4	0.88	(0.78)
REM L (min)	105.8 ± 60.3	99.3 ± 63.6	0.82	(0.62)	95.1 ± 45.1	96.4 ± 40.9	0.72	(0.53)	116.1 ± 59.8	104.1 ± 48.4	0.74	(0.62)
Wake (min)	123.5 ± 61.5	116.8 ± 71.4	0.77	(0.51)	130 ± 62.4	120.3 ± 54.6	0.90	(0.83)	114.4 ± 72.9	99.4 ± 65.3	0.89	(0.83)
N1 + N2 (min)	249.2 ± 75.4	234 ± 78.1	0.92	(0.82)	198.7 ± 50.1	210.9 ± 47.5	0.75	(0.59)	216.6 ± 56.6	226.2 ± 47.9	0.79	(0.69)
N3 (min)	56.8 ± 52.8	72.7 ± 31.6	0.59	(0.09)	75.8 ± 39.6	73.3 ± 27.3	0.67	(0.45)	72.1 ± 41.3	75.6 ± 29.9	0.63	(0.47)
NREM (min)	306 ± 62.4	306.7 ± 86.3	0.86	(0.70)	274.4 ± 49	284.3 ± 44.4	0.86	(0.77)	288.7 ± 62.2	301.9 ± 54	0.87	(0.80)
REM (min)	71 ± 31.1	77.1 ± 30.8	0.83	(0.64)	74 ± 23.1	73.8 ± 21.6	0.75	(0.59)	74.5 ± 29.8	76.3 ± 23.4	0.84	(0.76)

ICC_95%AAAvg_, 95% absolute agreement ICC; LowB, lower bound of ICC_95%AAAvg_. ICC cutoffs: 0–0.39: “poor” agreement; 0.40–0.59: “fair” agreement (yellow); 0.60–0.74: “good” agreement (light green); 0.75–1: “excellent” agreement (green) [15].

TST, total sleep time; SE, sleep efficiency; WASO, wake after sleep onset; SL, sleep latency; LPS, latency to persistent sleep; REML, REM sleep latency.

For the entire sample, the IRR, based on ICC values was “good” for N3 sleep and “excellent” for all remaining sleep parameters presented in Table 2. The healthy sub-group presents “excellent” ICC for TST, SE, WASO, SL, LPS, REML, and wake, “good” for N1 + N2 and REM sleep and “fair” for N3 and NREM sleep. For the overall pathology dataset, “excellent” ICC was observed for TST, SE, WASO, SL, LPS, REML, N1 + N2, and REM sleep and “good” ICC for N3 sleep. For OSA patients ICC of TST, WASO, REML, wake, N1+N2, NREM, and REM sleep was “excellent”, while SE, SL, and LPS had “good” ICC and N3 “fair” ICC. All the sleep parameters of the insomniac and MDD patients had “excellent” ICC, except REML and N3 with “good” ICC in both study groups.

**Figure 1.**
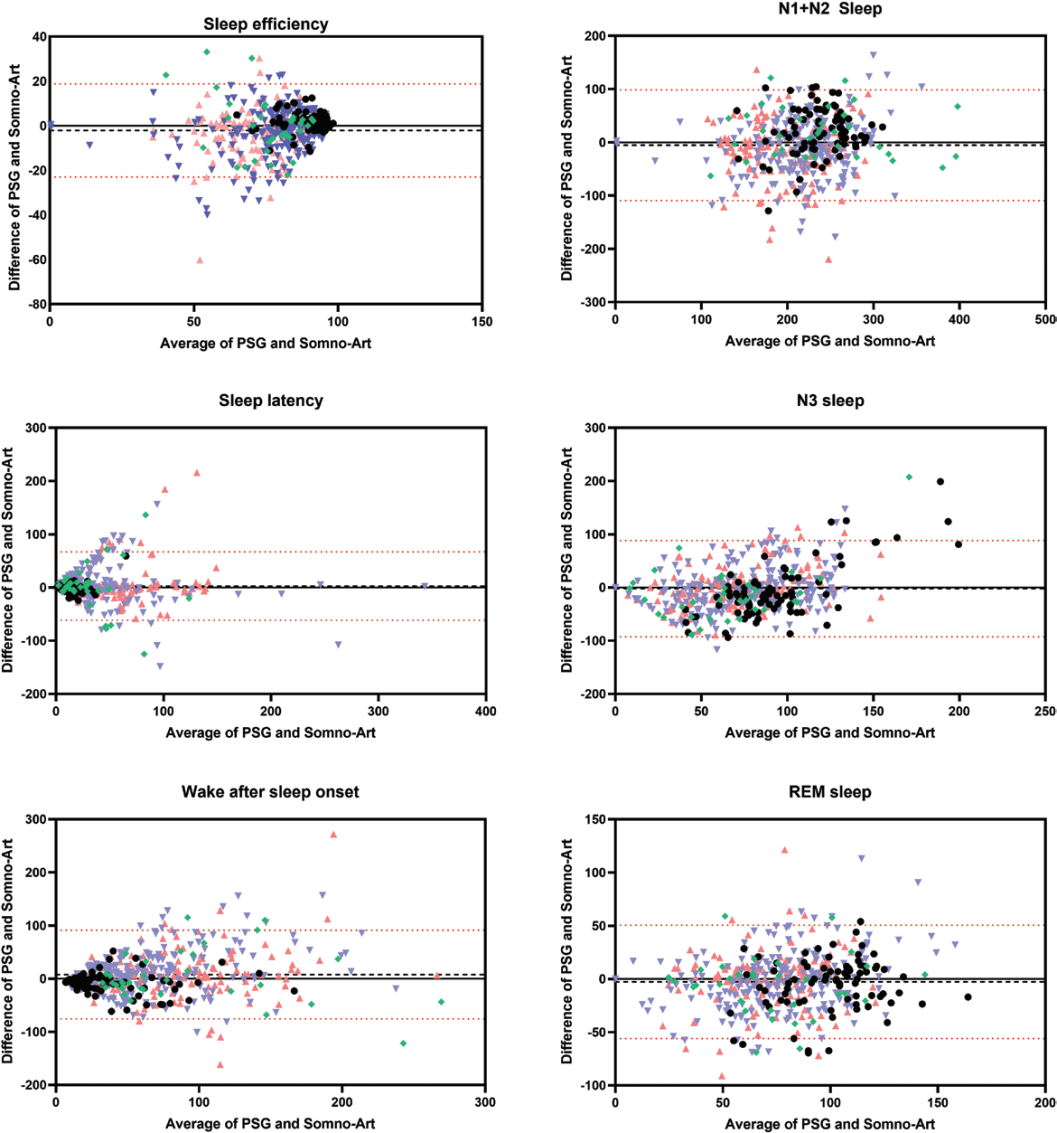
Bland-Altman plots for sleep efficiency, sleep latency, wake after sleep onset, N1 + N2, N3, and REM sleep. Bias and lower and upper LoA between PSG and Somno-Art Software of the overall group are represented (*n* = 458). Black dots represent the healthy group (*n* = 79), green diamond OSA patients (*n* = 33), pink upward triangle insomniac patients (*n* = 135), and blue downward triangle MDD patients (*n* = 211).

Bland-Altman plots (Figure 1) and the specific bias, ±95% CI of the biases and the lower and upper LoA (Table 3) show the trend for a possible under or overestimation of Somno-Art Software compared to visual scorer. On the overall group, Somno-Art Software overestimated SE by 2.07%, N1 + N2 by 5.24 min, N3 by 2.16 min, and REM sleep by ≤ 3 min (on a mean TST of 369 min), while SL and WASO were underestimated by less than 3 min and 8 min respectively.

**Table 3.** Bland-Altman plot biases, ±95% CI of the biases, lower and upper agreement limits for Somno-Art Software versus PSG in the overall group and in the sub-groups: healthy, pathologies, OSA, insomniac, and MDD patients

		Bias ± *SD*	±95% CI of the biases	Lower agreement limit	Upper agreement limit
SE (%)	All	−2.07 ± 0.63	−3.30 to −0.85	−22.98	18.83
	Healthy	1.68 ± 0.79	0.12 to 3.23	−7.27	10.62
	Pathologies	−2.52 ± 0.69	−3.87 to −1.17	−24.41	19.38
	OSA	−0.94 ± 2.40	−5.64 to 3.76	−26.86	24.99
	Insomniac	−2.04 ± 1.05	−4.10 to 0.01	−22.16	18.08
	MDD	−3.15 ± 0.92	−4.96 to 1.35	−24.89	18.59
SL (min)	All	2.51 ± 1.87	−1.16 to 6.17	−61.79	66.8
	Healthy	0.37 ± 1.45	−2.47 to 3.21	−18.89	19.62
	Pathologies	2.76 ± 2.09	−1.33 to 6.85	−65.60	71.12
	OSA	−0.18 ± 8.16	−16.17 to 15.82	−89.95	89.6
	Insomniac	4.90 ± 2.73	−0.45 to 10.25	−54.58	64.38
	MDD	2.33 ± 2.79	−3.15 to 7.80	−64.53	69.18
WASO (min)	All	7.69 ± 2.43	2.92 to 12.45	−75.64	91.01
	Healthy	−8.42 ± 3.01	−14.32 to −2.53	−45.94	29.1
	Pathologies	9.60 ± 2.67	4.37 to 14.83	−77.62	96.81
	OSA	6.91 ± 8.64	−10.03 to 23.85	−87.03	100.85
	Insomniac	4.81 ± 4.62	−4.25 to 13.87	−84.58	94.19
	MDD	12.83 ± 3.46	6.04 to 19.61	−71.18	96.83
N1 + N2 (min)	All	−5.24 ± 3.07	−11.26 to 0.79	−109.16	98.69
	Healthy	10.12 ± 8.23	−6.00 to 26.24	−81.62	101.86
	Pathologies	−7.05 ± 3.28	−13.48 to 0.62	−112.38	98.28
	OSA	15.25 ± 8.16	−0.74 to 31.24	−74.23	104.73
	Insomniac	−12.27 ± 6.12	−24.26 to −0.28	−123.07	98.52
	MDD	−9.67 ± 4.31	−18.11 to −1.23	−113.46	94.13
N3 (min)	All	−2.16 ± 2.82	−7.69 to 3.37	−92.53	88.2
	Healthy	8.57 ± 11.07	−13.12 to 30.27	−108.30	125.45
	Pathologies	−3.43 ± 2.87	−9.06 to 2.19	−89.92	83.05
	OSA	−15.9 ± 9.90	−35.30 to 3.50	−122.69	90.89
	Insomniac	2.43 ± 4.63	−6.64 to 11.49	−75.89	80.74
	MDD	−3.54 ± 3.74	−10.86 to 3.78	−88.14	81.07
REM (min)	All	−2.75 ± 1.54	−5.77 to 0.27	−56.02	50.52
	Healthy	−10.64 ± 4.29	−19.05 to −2.24	−62.04	40.75
	Pathologies	−1.82 ± 1.64	−5.03 to 1.40	−55.18	51.55
	OSA	−6.08 ± 5.07	−16.03 to 3.86	−61.20	49.03
	Insomniac	0.14 ± 2.99	−5.72 to 5.99	−54.51	54.79
	MDD	−1.83 ± 2.11	−5.96 to 2.31	−54.23	50.58

In healthy subjects, Somno-Art Software underestimated SE by ≤ 2%, N1+N2 by 10.12 min, N3 sleep by 8.57 min, and SL by < 1 min. Somno-Art Software overestimated REM sleep by 10.64 min and WASO by 8.42 min

In the patient group, SE was overestimated by 2.52%, N1+N2 sleep by 7.05 min, N3 sleep by 3.43 min, and REM sleep by <2 min. Somno-Art Software underestimated SL by 2.76 min and WASO by 9.60 min.

Table 4 illustrates EBE agreement measured with accuracy, κ coefficient, sensitivity, and specificity. Wake/sleep classification ranges from accuracy of 82.8% for the OSA sub-group to an accuracy of 93% for the healthy sub-group. κ coefficient for wake/sleep was moderate for OSA sub-group (κ: 0.54) and substantial for the other groups (κ: 0.63–0.70). Sensitivity ranges between 88.9% (OSA patients) to 95.1% for the healthy sub-group, while specificity was lowest for OSA patients with 64.5% and highest for insomniac patients with 74.5%. For the four stages classification (W/N1 + N2/N3/REM), accuracy was lowest for OSA patients with 63.9% and highest for healthy patients 71.2%. κ coefficient was moderate for all studied groups.

**Table 4.** Two stages accuracy, κ, sensitivity, and specificity and four stages accuracy and κ for the overall group and split by study groups

	Wake/Sleep Classification				W/N1+ N2/ N3/REM Classification	
	Accuracy	Kappa	Sensitivity	Specificity	Accuracy	Kappa
All (*n* = 458)	87.8%	0.65	93.3%	69.5%	68.5%	0.55
Healthy (*n* = 79)	93.0%	0.63	95.1%	73.3%	71.2%	0.57
Pathologies (*n* = 379)	86.7%	0.64	92.8%	69.2%	68.0%	0.54
OSA (*n* = 33)	82.8%	0.54	88.9%	64.5%	63.9%	0.46
Insomniac (*n* = 135)	87.7%	0.70	93.2%	74.5%	69.8%	0.57
Depressed (*n* = 211)	86.6%	0.62	93.2%	65.8%	67.5%	0.53

κ cutoffs: <0: poor; 0–0.20: slight; 0.21–0.40: fair; 0.41–0.60: moderate (yellow); 0.61–0.80: substantial (green); 0.81–1: almost perfect agreement [25].

The confusion matrices (Table 5) illustrate the percentage agreement between Somno-Art Software and PSG visual scoring for each sleep stage. For all study groups, confusions between Somno-Art Software and PSG are mostly due to N1 + N2 sleep misclassification and principally confusions with N3 sleep. Misclassification mean between wake and REM sleep was < 10%. Sleep stage accuracy across the various studied groups was >85% for wake, N3, and REM sleep, and between 68% and 73% for N1 + N2 sleep.

**Table 5.** Confusion matrices between Somno-Art Software and visual scoring of PSG for the different study groups

	All		Somno-Art Software				Accuracy		OSA		Somno-Art Software				Accuracy
			W	N 1+ N2	N3	REM					W	N1 + N2	N3	REM	
PSG	W	**(23.2%)**	**69.5%**	23.9%	1.9%	4.7%	87.8%	PSG	W	**(24.9%)**	**64.5%**	28.0%	3.2%	4.4%	83.1%
	N1 + N2	**(45.2%)**	9.3%	**69.0%**	12.7%	8.9%	71.5%		N1 + N2	**(49.5%)**	13.7%	**63.7%**	12.4%	10.2%	67.8%
	N3	**(15.5%)**	1.5%	32.7%	**64.1%**	1.7%	88.0%		N3	**(11.7%)**	4.9%	30.2%	**62.9%**	1.9%	88.6%
	REM	**(16.1%)**	4.6%	24.0%	1.4%	**70.1%**	89.8%		REM	**(13.9%)**	7.2%	28.1%	0.8%	**63.9%**	88.7%
	Healthy		Somno-Art Software				Accuracy		Insomniac		Somno-Art Software				Accuracy
			W	N1 + N2	N3	REM					W	N1 + N2	N3	REM	
PSG	W	**(9.6%)**	**73.3%**	21.8%	1.2%	3.7%	93.0%	PSG	W	**(29.6%)**	**74.5%**	19.9%	1.2%	4.4%	87.6%
	N1 + N2	**(51.4%)**	7.1%	**69.3%**	14.4%	9.2%	72.9%		N1 + N2	**(40.5%)**	9.7%	**69.4%**	12.0%	8.8%	72.8%
	N3	**(18.8%)**	1.2%	29.6%	**67.6%**	1.7%	86.3%		N3	**(15.1%)**	0.8%	33.6%	**63.7%**	2.0%	89.1%
	REM	**(20.2%)**	2.7%	18.2%	0.5%	**78.6%**	90.2%		REM	**(14.8%)**	5.1%	25.9%	1.5%	**67.5%**	90.0%
	Pathologies		Somno-Art Software				Accuracy		Depressed		Somno-Art Software				Accuracy
			W	N1+N2	N3	REM					W	N1 + N2	N3	REM	
PSG	W	**(26.0%)**	**69.2%**	24.1%	1.9%	4.8%	86.7%	PSG	W	**(23.9%)**	**65.8%**	26.8%	2.3%	5.2%	86.7%
	N1 + N2	**(43.9%)**	9.9%	**68.9%**	12.3%	8.9%	71.2%		N1 + N2	**(45.2%)**	9.3%	**69.6%**	12.5%	8.6%	70.8%
	N3	**(14.8%)**	1.6%	33.5%	**63.2%**	1.7%	88.4%		N3	**(15.2%)**	1.7%	33.9%	**63.0%**	1.4%	87.9%
	REM	**(15.2%)**	5.1%	25.6%	1.6%	**67.7%**	89.7%		REM	**(15.7%)**	4.8%	25.0%	1.8%	**68.3%**	89.7%

Values are normalized by row. Bold numbers correspond to agreement scores. Numbers under brackets correspond to the percentage of each sleep stage measured with PSG.

## Discussion

The results of the present study bring additional evidence for using algorithms that combine heart rate and body movement for scoring normal sleep in accordance to standard visual rules. The present research further extends these results to the 2 most common sleep disorders (chronic insomnia and OSA) as well as to sleep of patients with MDD.

When considering all the investigated sleep parameters (TST, SE, WASO, SL, LPS, REML, Wake, N1 + N2, N3, NREM, and REM sleep), with the exception for N3 sleep in healthy and OSA patients and NREM sleep in healthy subjects where the ICC were “fair”, the agreement between Somno-Art Software and PSG showed “excellent” or “good” ICC in healthy, OSA, insomniac, and MDD patients. Interestingly, healthy and OSA patients present higher bias for N3 sleep than the other groups. A closer look at the Bland-Altman plot shows that in the case of the healthy group, the bias increases with longer N3 duration: Somno-Art Software tends to underestimate N3 sleep for long N3 sleep durations (> 150 min). In OSA patients, who present with the lowest mean N3 sleep duration (56.8 min), Somno-Art Software tends to overestimate N3 sleep duration.

As expected from the sleep parameter analysis, EBE analysis achieves promising results. Sensitivity, specificity, accuracy, and κ coefficient of the overall dataset were 93.3%, 69.5%, 87.8%, and 0.65, respectively. Movement-based wearable devices, such as actimetry, often suffer from poor specificity, with difficulties detecting calm wake periods. A systematic review of the literature indicated that the specificity of actimetry ranges between 28% and 67% in healthy population [26]. Somno-Art Software showed a higher specificity compared to actimetry on healthy subjects (mean specificity: 73.3%) and even on recording nights from patient population (mean specificity: 69.2%). Compared to other algorithm based on heart rate and wrist movements, Somno-Art Software shows higher specificity in insomniac patients compared to the algorithm evaluated by Kahawage et al. (74.5% for Somno-Art Software vs. 45% by Kahawage et al. [27]), while Fonseca et al. [28] showed similar performances to the present results on a sample of patients with sleep disorders (69.2% for Somno-Art Software versus 72.9% for Fonseca et al. [28]).

On four stages classification (W/N1 + N2/N3/REM), Somno-Art Software presented an accuracy and κ coefficient of 68.5% and 0.55 respectively on the overall dataset, a performance comparable to the heart rate-based algorithm evaluated by Radha et al. [29] on a similar population: accuracy: 77%, κ: 0.61.

Sleep stage accuracy was > 85% for wake, N3, and REM sleep. These results are comparable or slightly above the IRR of visual scorers for wake and REM sleep, but clearly exceed it for N3 sleep [4]. Visual scorers present the highest inter-rater variability for the sleep stage N3, generally due to the complexity associated with the measurement of slow waves (SW) duration and amplitude. In contrast, Somno-Art Software, as an automatic algorithm, is consistent in its definition of SW and may therefore yield more accurate results. Of note, the interpretation of the confusion matrices is improved by taking the duration of the sleep stage into consideration. In the case of wake, which represent 23.2% of the scored recording for the overall dataset, 69.5% were correctly scored with the software, while 30.5% of waking episodes were misclassified as sleep. But in parallel, only 6.5% of sleep episodes, that represent 76.8% of the scoring, were misclassified as wake. Moreover, the overall accuracy of wake was 87.8%. To further illustrate this point, insomniac patients that have more wake epochs (29.6%) overall as compared to the other participant groups, present a higher wake sensitivity (74.5%). Similarly, N3 sleep represents only 15.5% of the scored recording for the overall data set, and in consequence presents the lowest score with only 64.1% epochs correctly scored. However, the overall accuracy of N3 sleep is at 88.0%.

Somno-Art Software presents an average accuracy of 71.5% for the discrimination of N1 + N2 sleep on the overall dataset. Most misclassifications were observed between N1 + N2 and N3 sleep. This finding is not surprising as N1 + N2 sleep represent the predominant sleep stage. Moreover, N1 sleep stage has low inter-rater agreement even between human scorers [2, 4, 17, 30], and as previously mentioned, confusion between N2 and N3 sleep stages is well known for visual scoring [4], as the characterization of N3 sleep depends on the amount of SW also present in N2 sleep [1].

**Figure 2.**
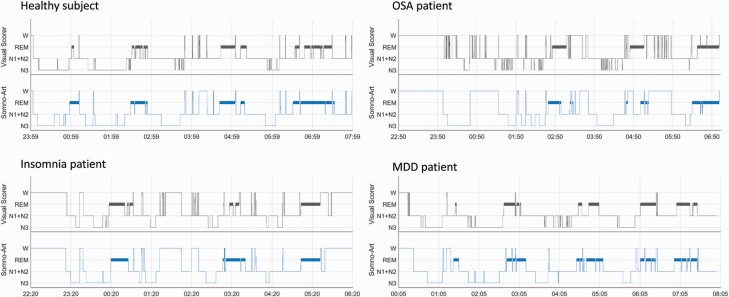
Example of hypnograms obtained with PSG (in black) and Somno-Art Software (in blue) for a healthy subject, an obstructive sleep apnea (OSA), an insomniac and a MDD patient. W, wake.

Figure 2 exhibits examples of hypnograms obtained with PSG and Somno-Art Software for healthy subjects, OSA, insomniac, and MDD patients. Even if the sleep cycles and the sleep structure between N1 + N2, N3, and REM sleep are preserved with Somno-Art Software for these four examples of sleep profiles, Somno-Art Software’s hypnograms are less fragmented and switches between N1 + N2 sleep and N3 sleep are less frequent compared to PSG, illustrating the lower performances of Somno-Art Software in the estimation of N1 + N2 sleep.

Interestingly, these results indicate comparable scoring performances of the Somno-Art Software in normal and pathological sleep. It should be emphasized that, in contrast to the results obtained with the Somno-Art Software, scoring pathological sleep has consistently been found less reliable than scoring normal sleep [2, 16, 17]. Indeed, sleep of patients often presents a fragmented hypnogram and less obvious sleep stages characteristics (K-complex, spindles, SW) than healthy subjects, leading to higher variance in visual sleep scoring. In addition, Rechtschaffen and Kales and subsequently the AASM sleep scoring rules have been developed for healthy individuals and may not adequately describe disturbed sleep [2]. Cardiac-based sleep scoring algorithms are usually evaluated only in healthy subjects [14, 19, 20, 31, 32]. Fortunately, recent studies are starting to evaluate algorithms on patients suffering from sleep disorders [27–29] which is necessary to ascertain whether they fit with data coming from patients with disrupted sympatho-vagal balance, such as patients with OSA, insomnia, or MDD [33–35].

## Limitations

Of note, the difference in the sample size of each study group may lead to different statistical power and thus different levels of precision. However, this concern is less relevant due to the stated objective of this study which is to evaluate the performance of the software on the various study groups and was not intended for a between groups comparison.

The analyzed data were already integrated in the learning process of the algorithm and could present a bias in the performances of the outcome of analysis. However, software performances have been trained on large datasets (more than 600 nights, including data used for above results) and the technologies used for this sleep classification algorithm are resistant to overfitting.

One current limitation of the ongoing version of Somno-Art Software is the duration of the recording. Indeed, the Software has been validated on recordings longer than 5 h and is therefore currently inadequate for use with shorter recordings (e.g. nap).

## Conclusion

The present study indicates that Somno-Art is a reliable tool for the characterization of sleep architecture and continuity in both healthy subjects and patients with OSA, insomnia, or MDD. It opens new insights to measure sleep at home, in a less invasive and costly, and more time-saving way than the gold standard, PSG. Somno-Art could for instance complement existing non-attended techniques measuring sleep-related breathing pattern or be a useful alternative to laboratory-based PSG when this latter is not available.

## Supplementary Material

zpab019_suppl_Supplementary_Data
