# Supplementary material for: Validation of Somno-Art Software, a novel approach of sleep staging, compared with polysomnography in disturbed sleep profiles
Source: Sleep Adv. 2021 Dec 9;3(1):zpab019. doi: 10.1093/sleepadvances/zpab019 (PMC10104381; doi:10.1093/sleepadvances/zpab019)
Supplement: zpab019_suppl_Supplementary_Data [file zpab019_suppl_supplementary_data.docx]

**Validation of Somno-Art Software, a novel approach of sleep staging, compared with polysomnography in disturbed sleep profiles**

Laurie Thiesse^1^, Luc Staner^2^, Patrice Bourgin^3^, Thomas Roth^4^, Gil Fuchs^1^, Debora Kirscher^1^, Jean Yves Schaffhauser^1^, Jay Saoud^5,6^, Antoine U. Viola^1^

*1. PPRS, Colmar, France*

*2. Unité d’exploration des rythmes veille sommeil, hospital Rouffach, France*

*3.*  *Sleep Disorders Center & CIRCSom (International Research Center for ChronoSomnology), Strasbourg University Hospital, Strasbourg, France; CNRS UPR 3212, Institute for Cellular and Integrative Neurosciences, Strasbourg, France.*

*4.* *Sleep Disorders Center, Henry Ford Hospital, Detroit, MI, USA*

*5. PPRS Research Inc., Massachusetts, USA*

*6.* *PPDA, LLC, Massachusetts, USA*

**Corresponding Author:**

Antoine Viola

PPRS

4E Avenue du General de Gaulle

68000 Colmar

France

avi@pprs-research.com

**Supplementary 1**

- **Protocol description “Healthy study 1“:**

The primary objective of the healthy study 1, performed in 2012, was to investigate the relationship between the spontaneous and controlled activity during the day and the sleep structure overnight as well as the impact of noise on the sleep pattern in healthy young male and female volunteers.

After a screening period, subjects spent five consecutive nights in the sleep center with PSG, holter-ECG and actimetry recording. The first night was an adaptation night, followed by two days with various physical activity exercices. During the fourth night, subjects were exposed to recorded motor-vehicule and aircraft noises. The disturbed night was followed by a last recovery night. Subjects had a 8h sleep opportunity per night with a light off between 22:00 and 00:00 and a light on between 6:00 and 8:00.

Only healthy male and females between 18 and 40 years were studied.

- **Protocol description “Healthy study 2”:**

The healthy study 2 is a monocentric, randomized, crossed- comparative study performed between 2014 and 2019. The primary aim of the study was to investigate the direct effect of blue light on sleep, wake, EEG and cognitive performances in relation to sleep pressure and the circadian timing.

The study started with a recruitment phase with various questionnaires to assess sleep quality, chronotype, depression and insomnia scale (Medical Outcome Study Short Form-36 (SF36), Horne-Ostberg (HO), Munich ChronoType Questionnaire (MCTQ), Pittsburgh Sleep Quality Index (PSQI), Epworth sleepiness scale (ESS), Hamilton Rating Scale for Depression (HDRS), Snaith-Hamilton pleasure scale (SHAPS), Insomnia severity index (ISI) questionnaire).

During the second phase which took place one week prior the study, the sleep and circadian pattern of the volunteers were recorded using an actimeter and a sleep log journal. The study *per se* started with a screening night to adapt the subjects to the sleep center environment and detect possible sleep disorders. The selected volunteers had a mean sleep duration of 8 hours. The study was composed of two circadian protocols: two 40 hours constant routine to measure endogen circadian rhythm (with blue or white light) and two 40 hours nap protocols (with blue or white light). The order of the four sessions was randomized and separated each other by 1 to 8 weeks. Each session started with a baseline night of 8 hours sleep followed by a sleep deprivation of 40 hours or a 40 hours nap protocol and ending with a recovery night of 8hours. PSG, including an ECG lead as well as actimetry were continuously assessed during the study.

- **Protocol description “OSA study”:**

Since 2014 a multicentric, non-pharmacological, non-randomized and non-controlled study aims at evaluating sleep architecture of patients admitted in a sleep exploratory unit using Somno-Art in comparison to PSG.

Included patients were male and female adults suffering from sleep disturbances as classified by the ICSD-2: insomnia, respiratory sleep disorders, central hypersomnia, circadian rhythm disorder, parasomnia, movement sleep disorders, isolated symptoms and other disorders, for which a PSG examination is indicated.

After a first visit during which the inclusion reason was determined, patients were recorded for 1 to 3 consecutive nights with PSG and actimetry in the sleep unit.

- **Protocol description “Insomniac study”:**

A randomized, placebo-controlled, 2-way crossover, double-blind study has been enrolled in 2015 to evaluate the efficacy, safety and tolerability of a drug candidate in subjects with insomnia disorder without psychiatric comorbidity.

The study started with an eligibility screening examination: a general health assessment (on the basis of clinical laboratory tests, medical history, vital signs and 12-lead ECG performed at screening) and subjective insomnia (ISI questionnaire, report difficulty with sleep onset and sleep maintenance) and an objective assessment of insomnia with three consecutive PSG screening nights in the sleep centre (subjects had to meet the following objective inclusion criteria: 2-night mean latency to persistent sleep ≥30 minutes with no night <20 minutes, and one of both nights with TST ≤6 hours and WASO>30 minutes). Subjects with restless leg syndrome/periodic limb movements, apnea, parasomnias or other sleep disorders were excluded on the first screening night.

The double-blind crossover treatment phase consists of two treatment periods of five consecutive days separated by a wash out period of at least five days and maximum nine days. Subjects were randomly assigned to one of the two treatment sequences (placebo-treatment or treatment-placebo). Actimetry and PSG were recorded on the first and last study night over 8 hours.

- **Protocol description “Depression study 1”:**

A multi-centre, double-blind, diphenhydramine and placebo-controlled study has been enrolled in 2015 to evaluate the safety, efficacy and biomarker of a drug candidate in subjects with major depressive disorders (MDD).

The study started with an eligibility screening examination: the symptoms of depression were assessed using the structured interview guide for the Hamilton Depression Scale (SIGHD-IDS), and Inventory of Depressive Symptomatology-clinician rated 30 (IDS-C30) (combined in the SIGHD-IDS) and the self-rated Quick Inventory of Depressive Symptoms-16 (QIDS-SR16). Subjects were excluded if they took hypnotics. Anti-Depressants (SSRI and TCA) were allowed. After this first check, subjects underwent two consecutive 8h PSG and actimetry recording nights in the clinic.

The double-blind treatment period was designed as follows. On Day 1, subjects were randomly assigned (in a 2:1:1 ratio) to either the drug candidate or diphenhydramine or placebo q.d. in the evening over 10 days (women of childbearing potential (WOCBP)) or 4 weeks (males and women of non-childbearing potential (WONCBP)). Randomization was stratified for males/WONCBP or WOCBP. Males and WONCBP took 1 capsule every evening just before bedtime from Day 1 to Day 28 and from Day 1 to Day 10 for WOCBP. PSG and actimetry were recorded on day 1, 5 and 10 for 8 hours. On night 5, a forced nighttime awakening was realized one hour and 30 minutes after dosing to assess cognitive and psychomotor functions, attention, short and long-term memory and assess plasma concentration of the drug.

- **Protocol description “Depression study 2”:**

A randomized, double-blind, parallel-group, placebo- and active-controlled study has been enrolled in 2015 to evaluate the efficacy and safety of a drug candidate in adults with MDD. Male and female meeting the DSM-5 diagnostic criteria for moderate to severe MDD, without psychotic features, were studied. The study started with a screening period, among other to assess depression symptoms with Montgomery-Asberg Depression Rating Scale (MADRS) and allowing a washout of previous medications and a baseline visit two days before the second phase. Subjects were excluded if they use psychotropic drugs. They were allowed to take the following medication: analgesics, antacids, antiasthma agents, antibiotics, anticoagulant, antidiarrheal preparations, antifungal agents topical, antihistamine, antinauseants, cough/cold prep, diuretics, H2 blockers, hormones.During the baseline night sleep was recorded with PSG and actimetry. The study was followed by a double-blind, placebo-and active controlled treatment phase where sleep was recorded with PSG and actimetry at the beginning (day 1), the middle (day 15) and the end of the phase (day 43).
